# Induction of Monozygotic Twinning by Ascorbic Acid in Tobacco

**DOI:** 10.1371/journal.pone.0039147

**Published:** 2012-06-18

**Authors:** Zhong Chen, Daniel R. Gallie

**Affiliations:** Department of Biochemistry, University of California, Riverside, Riverside, California, United States of America; Ben-Gurion University of the Negev, Israel

## Abstract

Embryo development in plants initiates following the transverse division of a zygote into an apical, proembryo cell and a basal cell that gives rise to the suspensor. Although mutants affected in embryo development through changes in cell division have been described, little is known about the control of the first zygotic division that gives rise to the proembryo. Ascorbic acid (Asc) promotes cell division by inducing G_1_ to S progression but its role in embryo development has not been examined. In this study, we show that the level of dehydroascorbate reductase (DHAR) expression, which recycles Asc and regulates Asc pool size, affects the rate of monozygotic twinning and polycotyly. DHAR-induced twinning resulted from altered cell polarity and longitudinal instead of transverse cell division that generated embryos of equal size. Direct injection of Asc into ovaries phenocopied DHAR-induced twinning. Twinning induced by Asc was developmentally limited to the first two days after pollination whereas polycotyly was induced when the level of Asc was elevated just prior to cotyledon initiation. This work describes the first example of gene-directed monozygotic twinning and shows that Asc regulates cell polarity during embryo development.

## Introduction

Embryo development initiates following the transverse division of a zygote into an apical, proembryo cell and a basal cell that gives rise to the suspensor, to generate one embryo per seed. The presence of more than one embryo per seed can result from apomixis, which occurs in a wide range of flowering plant taxa, or by twinning [1]–[3], [4]. Apomixis is the asexual formation of a seed from maternal ovule tissues not involving meiosis and fertilization which results in monoembryony or polyembryony. In apomicts, embryos can arise spontaneously from ovule cells (i.e., sporophytic apomixis) or from cells of an unreduced embryo sac (i.e., gametophytic apomixis) depending on the species [4]. In either case, embryos resulting from apomixis are genetically maternally-derived. Twinning commonly refers to the generation of more than one zygotically-derived embryo following fertilization. Identical twins arise from the same zygote, either following the first zygotic division (i.e., monozygotic twinning) or ectopically from other embryonic tissues such as the suspensor [5]–[7].

In Arabidopsis, embryos of the *twn2* mutant exhibit reduced valyl-tRNA synthetase expression resulting in growth arrest of the embryo proper and embryogenetic transformation of suspensor cells [6]. Embryogenetic development of suspensor cells also occurs in the *twn1* and *amp1* mutants in the absence of embryo proper arrest, suggesting loss of embryo proper-suspensor communication [5]–[7]. In these examples, twinning resulted from embryo development from suspensor cells once suppression of the embryogenetic potential of the suspensor was released. In such mutants, the development of ectopic embryos requires changes in the plane of cell division within the suspensor (e.g., longitudinal instead of transverse) that enable the growth of embryos or embryoid tissue. These mutants also exhibited cotyledon fusion or polycotyly independent of suspensor transformation, suggesting that these genes can affect cotyledon development as well as embryo development. Despite the isolation of several mutants affecting embryo development, no mutant affecting the first zygotic division in a way that results in monozygotic twinning has been reported and therefore those factors controlling the correct formation of the apical, proembryo cell and basal cell are unknown.

Ascorbic acid (Asc) is a major antioxidant that serves several functions in plants. Asc is involved in the detoxification of reactive oxygen species, e.g., superoxide, singlet oxygen, ozone, and H_2_O_2_, which are produced during aerobic metabolic processes such as photosynthesis or respiration). Asc is required for the re [8] generation of α-tocopherol (vitamin E) from the tocopheroxyl radical [9]. Asc serves as a co-factor for enzymes such as violaxanthin de-epoxidase (VDE) in the xanthophyll cycle, ACC oxidase which generates ethylene, and prolyl and lysyl hydrolases [10]–[12] as well as for 2-oxoacid-dependent dioxygenases required for the synthesis of abscisic acid and gibberellic acid [13]–[15].

Asc may also be involved in regulating cell growth where it promotes the G1 to S phase progression of cells within the quiescent center (QC) of onion roots [16]–[20]. Asc is present in low amounts in the QC of maize roots and increases considerably during logarithmic growth of tobacco cell culture [21], [22]. Asc also reversed the inhibition of cell division caused by lycorine treatment which reduces Asc content [19].

Once used, Asc is oxidized to the monodehydroascorbate radical (MDHA) that can be reduced to Asc in the chloroplast stroma by either monodehydroascorbate reductase (MDHAR) or ferredoxin or in the cytosol by MDHAR [23], [24]. MDHA can also rapidly disproportionate to Asc and dehydroascorbate (DHA) [24]. DHA is then reduced to Asc by dehydroascorbate reductase (DHAR) in a reaction requiring glutathione. In the absence of sufficient DHAR, however, DHA undergoes irreversible hydrolysis to 2,3-diketogulonic acid. Because the apoplast contains little glutathione or DHAR, DHA, which predominates in the apoplast, is actively transported into the cell for reduction to Asc to avoid its hydrolysis.

Despite the several pathways in which Asc can be regenerated, the level of DHAR expression in leaves is rate-limiting for Asc recycling and therefore, DHAR significantly contributes to the control of the Asc pool size and redox state [25]–[28]. Increased DHAR expression improved the efficiency of Asc recycling resulting in a larger cytosolic and apoplastic Asc pool size and higher redox state (i.e., was more reduced) [25]–[28]. Conversely, suppression of DHAR expression reduced the efficiency of Asc recycling which resulted in a lower cytosolic and apoplastic Asc redox state (i.e., was more oxidized) [26]–[28].

Because Asc has been reported to promote cell division, we examined whether Asc regulates cell division during embryo development. We observed that a high level of DHAR expression induced monozygotic twinning and polycotyly. Twinning induced by DHAR resulted from altered cell polarity and longitudinal instead of transverse cell division of the zygote, generating embryos of equal size. Direct injection of Asc into ovaries phenocopied the DHAR-induced twinning demonstrating that it was the product of DHAR activity that was responsible for altered embryo development. Monozygotic twinning induced by Asc was developmentally limited to the first two days after pollination, consistent with its role in altering zygotic division. Similarly, polycotyly was induced when Asc levels were elevated just prior to cotyledon initiation. Our results demonstrate that the level of DHAR expression, and therefore, the level of Asc regulates cell polarity during embryo development.

## Results

### DHAR expression regulates polyembryony

**Figure 1:**
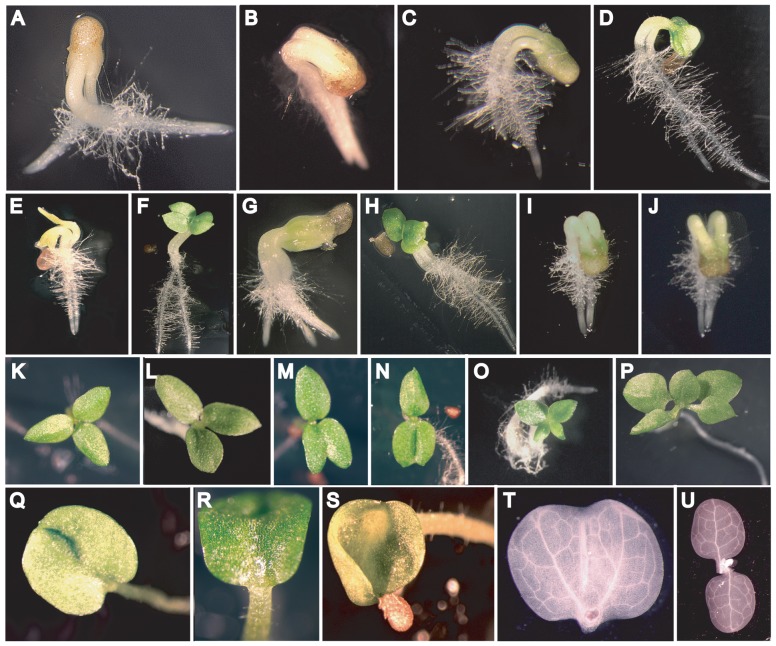
Ascorbate-induced twinning and polycotyly. **A–F**, Twin progeny emerging from DHAR-overexpressing tobacco seed. **G–H**, Triplet progeny emerging from DHAR-overexpressing tobacco seed. **I–J**, Twin progeny emerging from WT seed that developed from ovaries injected with 10 mM Asc on the day of pollination. **K–N**, Tricotyledon progeny from DHAR-overexpressing tobacco seed in various states of fusion. **O–P**, Tetracotyledon progeny from DHAR-overexpressing tobacco seed. **Q–T**, Monocotyledon progeny from DHAR-overexpressing tobacco seed where (**T**) the fused cotyledon was cleared of chlorophyll to reveal two main veins and plane of fusion. **U**, Wild-type seedling cleared of chlorophyll.

In previous work, we reported the generation of tobacco (i.e., *Nicotiana tabacum*) in which DHAR was overexpressed or suppressed [25]–[28]. To overexpress DHAR, tobacco was transformed with a wheat (i.e., *Triticum aestivum*) DHAR cDNA (D_Ta_) under the control of the cauliflower mosaic virus (CaMV) 35S promoter [25]. As D_Ta_ has diverged sufficiently from the tobacco DHAR ortholog at the nucleotide level, this approach avoided RNA silencing of DHAR expression. To suppress DHAR, tobacco was transformed with a tobacco DHAR cDNA (D_Nt_) under the control of the 35S promoter, resulting in the generation of lines in which DHAR expression was suppressed [26]. An increase in DHAR expression results in a larger Asc pool size [28]. Polyembryony (i.e., 2–3 embryos per seed)(Fig. 1A–H) and either polycotyly (3–4 cotyledons per seedling, Fig. 1K–P) or monocotyly (Fig. 1Q–S) were observed in D_Ta_ progeny at a higher frequency (9/4081 and 117/4081 seeds, respectively) than in vector-only lines (0/1774 and 9/1774 seeds, respectively) or lines expressing inactive, C-terminally-truncated, inactive D_Ta_ [26] (0/1496 and 3/1496 seeds, respectively). Removal of chlorophyll from seedlings exhibiting monocotyly revealed two main veins and a fusion plane that extended to the base (Fig. 1T) indicating fusion of two cotyledons that was also observed in tricot seedlings (Fig. 1M–N). The simple venation of the cotyledons in single and polycot seedlings and the observation that they arose from the first node (Fig. 1Q–T) indicated that they were true cotyledons. Cotyledon defects were as likely to result from monoembryonic D_Ta_ seed as from polyembryonic D_Ta_ seed, suggesting that polycotyly was not a consequence of polyembryony. Seed from DHAR-overexpressing tobacco were viable and twin seedlings grew normally with no vegetative defects, demonstrating that this aspect of DHAR overexpression was embryo-specific.

**Figure 2:**
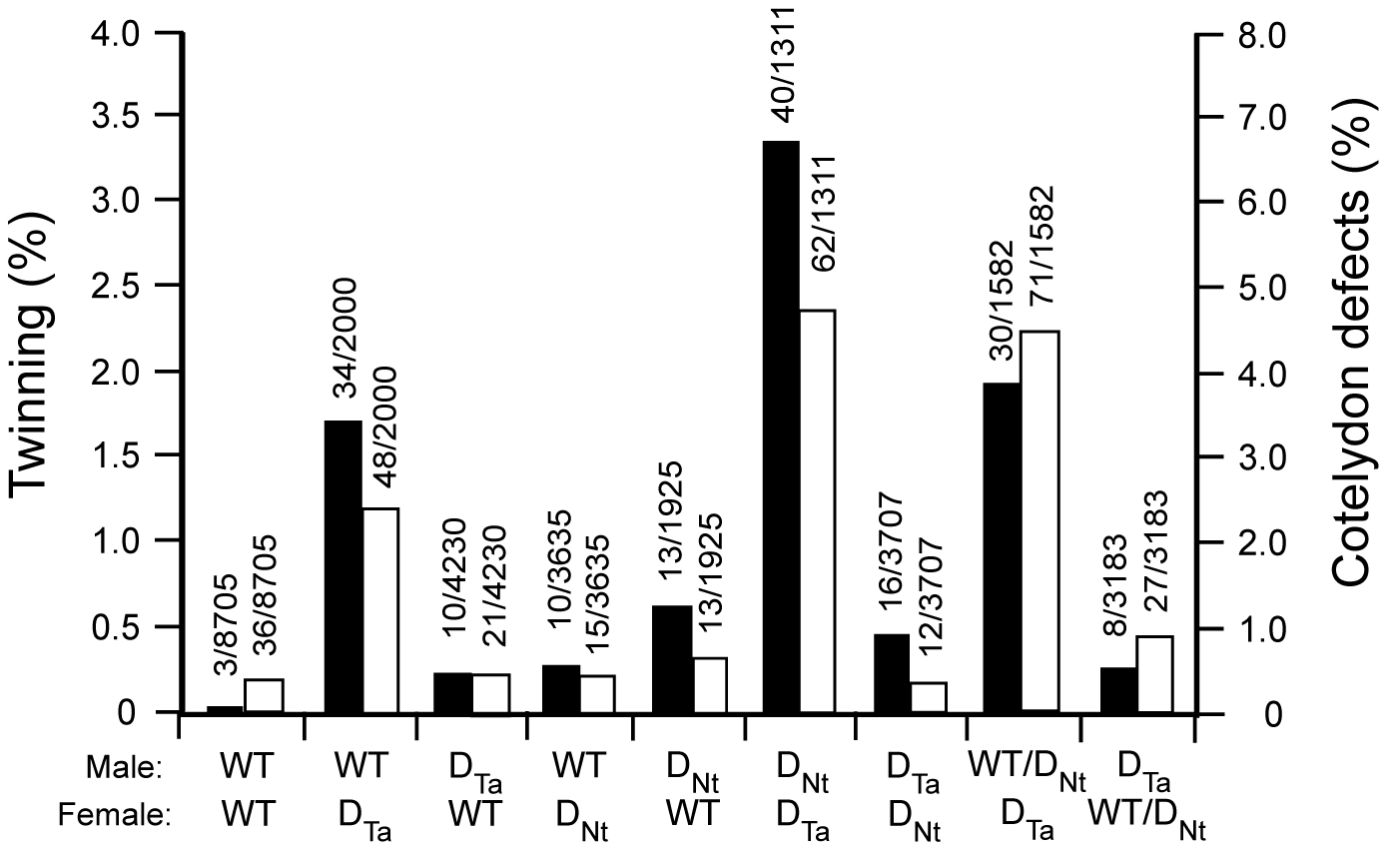
Frequency of ascorbate-induced twinning and polycotyly. Frequency of twinning and polycotyly resulting from crosses between WT and DHAR-overexpressing tobacco. Crosses between wild-type (WT) and tobacco transformed with wheat DHAR (D_Ta_) or tobacco DHAR (D_Nt_) were performed. The histograms represent the frequency of twinning (black bars, left scale) and cotyledon abnormalities (i.e., polycotyly or single fused cotyledon)(white bars, right scale) measured in the resulting seed.

To determine whether twinning results from inheritance of the DHAR_Ta_ transgene through the female or male germline, reciprocal crosses between wild-type (WT) and D_Ta_ plants were performed. An increase in twinning following DHAR_Ta_ transgene inheritance through male gametes would demonstrate that expression of DHAR in maternal tissues is not essential. Conversely, twinning resulting only from DHAR_Ta_ transgene inheritance through the female germline would suggest that transmission through male gametes is insufficient to cause twinning. An equal increase in twinning regardless of which parent transmits the DHAR_Ta_ transgene would suggest that twinning is dependent on inheritance of the DHAR_Ta_ transgene alone and that maternal effects are not involved. Progeny from a cross between a WT male (WT-m) and a D_Ta_ female (D_Ta_-f) exhibited a 50-fold increase in polyembryony (1.70%) relative to WT plants (0.034%)(Fig. 2), demonstrating that inheritance of the DHAR_Ta_ transgene through the female germline is sufficient to cause twinning. Progeny from a cross between a D_Ta_ male (D_Ta_-m) and a WT-female (WT-f) exhibited a 7-fold increase in polyembryony (0.24%) compared to WT plants (Fig. 2), demonstrating that inheritance of the DHAR_Ta_ transgene through the male germline is also sufficient to cause twinning but to a lower extent than in the reciprocal cross. These results demonstrate that maternal expression of DHAR, which results in an increase in the Asc pool size throughout the plant [28], induces a higher level of polyembryony than does paternal expression of DHAR. The polyembryony resulting from transmission of the D_Ta_ transgene through male gametes, however, demonstrates that DHAR-mediated polyembryony occurs after fertilization.

**Figure 3:**
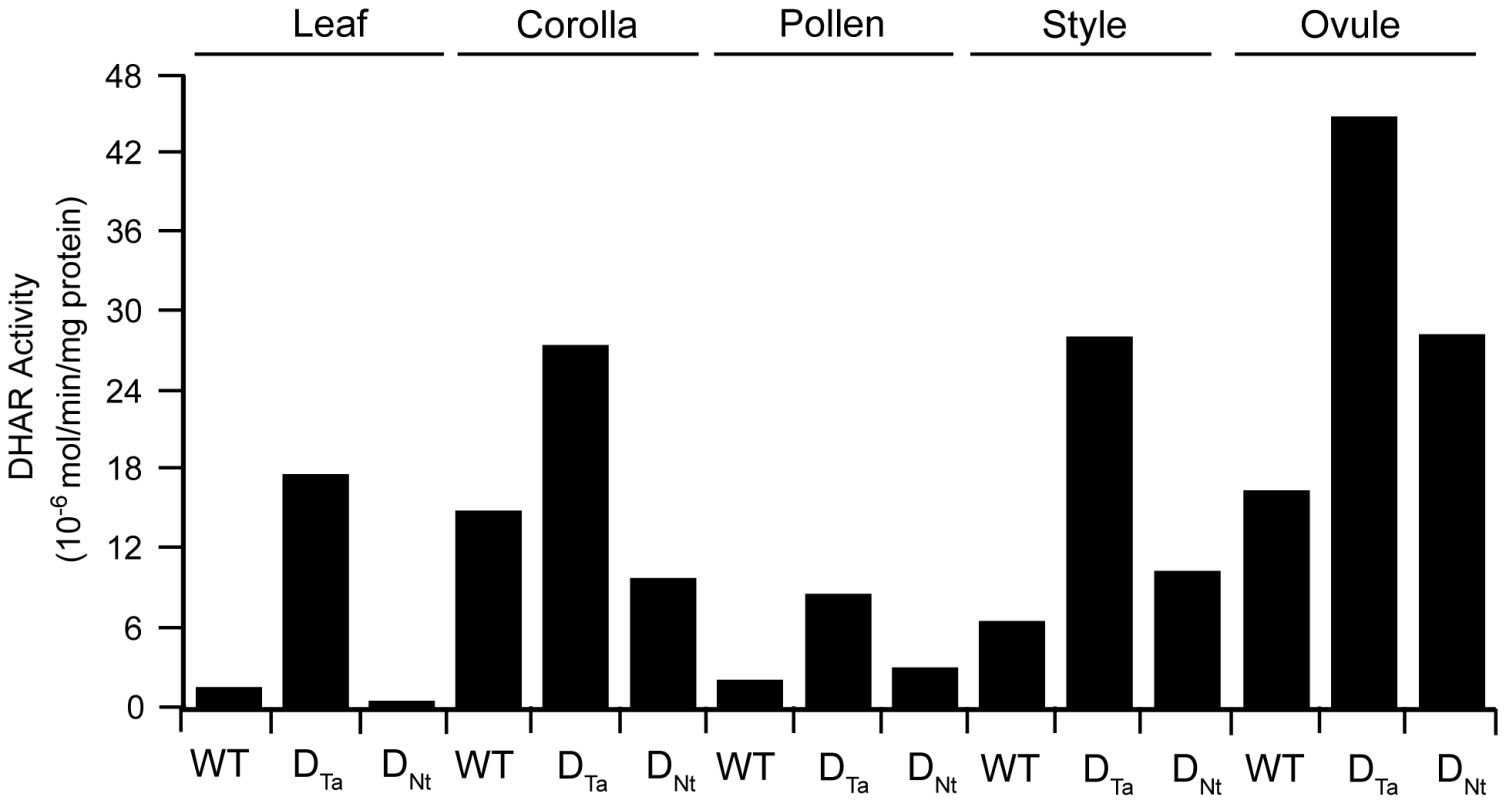
DHAR activity in leaves and floral organs of D_Ta_, D_Nt_, and WT plants. DHAR activity was measured in leaves or the indicated floral organs in DHAR-overexpressing (D_Ta_), DHAR-overexpressing (D_Nt_), and wild-type (WT) plants.

Overexpression of the D_Ta_ transgene results in an increase in DHAR expression throughout the plant (Fig. 3) whereas expression of the D_Nt_ transgene results in silencing of DHAR expression in leaves but not in ovules and results in a lower foliar Asc pool size and redox state during plant growth [28]. Measurements of Asc revealed that its level in DHAR_Nt_ ovules was lower (i.e., 0.861 µg/g FW) than in WT ovules (i.e., 1.25 µg/g FW) whereas the level of Asc in DHAR_Ta_ ovules was higher (i.e., 1.48 µg/g FW). Because of this, it was possible to separate maternal effects of Asc from Asc produced in the zygote specifically. The frequency of polyembryony was lower in a cross between a WT-m and a D_Nt_ female (D_Nt_-f) (0.28%) than between a WT-m and a D_Ta_-f (1.70%), demonstrating that increasing DHAR expression throughout the plant increases polyembryony. As Asc can be transported to sink tissues including floral organs [29], these results indicate that the frequency of polyembryony is likely determined by contributions made by maternal tissues in addition to embryo-derived Asc. The frequency of polyembryony in the cross between a WT-m and a D_Nt_-f was also lower than it was in a cross between a D_Nt_ male (D_Nt_-m) and a WT-f (0.68%), the reverse of the parental influence observed in reciprocal crosses between WT and D_Ta_ plants (Fig. 2). Therefore, the frequency of polyembryony was highest in crosses using a D_Ta_ female, followed by crosses using a WT female (when the DHAR transgene was provided by the male), which in turn was higher than in crosses using a D_Nt_ female. Because the Asc pool size is higher in D_Ta_ plants than it is in WT plants which in turn is higher in than it is in D_Nt_ plants (Chen and Gallie, 2006), these data support the conclusion that the maternal contribution of Asc influences the frequency of polyembryony. The frequency of polyembryony in the cross between a WT-m and a D_Nt_ female (D_Nt_-f), however, was higher (0.28%) than in WT plants (0.034%) despite the fact that the level of Asc is higher in WT plants than in D_Nt_ plants. As it is the level of Asc in the fertilized egg specifically that should determine the level of twinning, this observation suggests that the D_Nt_ transgene in the embryo is sufficient to modestly increase polyembryony even when the level of Asc in surrounding maternal tissues is low.

In reciprocal crosses between D_Ta_ and D_Nt_ plants, progeny from a cross between a D_Nt_-m and a D_Ta_-f exhibited a higher level of polyembryony (3.36%) than progeny resulting from a cross between a D_Ta_-m and a D_Nt_-f (0.43%)(Fig. 2), consistent with the previous conclusion that increasing the maternal Asc pool size substantially increases polyembryony. Interestingly, transmission of the D_Nt_ transgene through male gametes in the cross between a D_Nt_-m and a D_Ta_-f increased polyembryony approximately 2-fold more (3.36%) relative to a cross between a WT-m and a D_Ta_-f (1.70%), suggesting that the D_Nt_ and D_Ta_ transgenes each contribute to polyembryony and that the D_Nt_ transgene does so following its transmission by the male gametes. Compared to the level of polyembryony observed in the cross between a D_Nt_-m and a D_Ta_-f, the level of polyembryony was reduced almost 2-fold in a cross between a hemizygous D_Nt_-m (WT/D_Nt_-m) and a D_Ta_-f (1.9%)(Fig. 2), supporting the notion that transmission of the D_Nt_ transgene through male gametes contributes to polyembryony and reducing its presence in half of the male gametes results in a corresponding reduction in polyembryony. An increase in polyembryony was also observed in a cross between a D_Ta_-m and a D_Nt_-f (0.43%) relative to a cross between a WT-m and a D_Nt_-f (0.28%), supporting the conclusion that both transgenes contribute to polyembryony regardless of their transmission through male or female gametes. A reduction in polyembryony was observed in a cross between a D_Ta_-m and a hemizygous D_Nt_-f (WT/D_Nt_-f) (0.25%) relative to the cross between a D_Ta_-m and a homozygous D_Nt_-f (0.43%), additional evidence indicating that reducing the presence of the D_Nt_ transgene in half of the ovules results in a corresponding reduction in polyembryony.

Although the level of polycotyly also increased, this was largely limited to those crosses in which the D_Ta_ transgene was expressed in the female (Fig. 2). For example, relative to the level of polycotyly in wild-type plants (0.41%), a nearly 6-fold increase in polycotyly was observed in progeny from a cross between a WT-m and a D_Ta_-f (2.4%) but only a slight increase was observed in progeny from the reciprocal cross (0.5%)(Fig. 2). No increase in polycotyly was observed in progeny from a cross between a WT-m and a D_Nt_-f (0.41%)(where the female has a reduced Asc pool size in vegetative tissues) and only a slight increase in the reciprocal cross (0.68)(Fig. 2). A substantial increase in polycotyly was observed in progeny from a cross between a D_Nt_-m and a D_Ta_-f (4.73%) but no increase in progeny from the reciprocal cross (0.32%)(Fig. 2). These results suggest that it is not so much the presence of the DHAR transgene in the developing embryo but rather the maternal Asc pool size that most influences the frequency of polycotyly. However, transmission of a DHAR transgene through male gametes can contribute moderately to polycotyly especially in females expressing the D_Ta_ transgene.

Activity from the CaMV 35S promoter in developing embryos is not detected until the early heart stage [30]–[32]. Significantly higher expression of DHAR was observed in the reproductive organs of D_Ta_ and D_Nt_ plants, including in pollen, ovules, and style (Fig. 3). If twinning results from an increase in Asc, the observed twinning in D_Ta_ plants can be understood by the increase of DHAR expression in maternal tissues and transport of Asc to developing embryos [29]. Maternally-generated Asc can not explain, however, the twinning observed following fertilization of WT ovules by D_Nt_ pollen (Fig. 2). Although some studies have reported that the CaMV 35S promoter is silent in pollen, others have observed substantial activity, e.g., in strawberry, cotton, and pine [32]–[34]. Differences in the regulatory elements included in the CaMV 35S promoter used as well as the species examined may account for differences in the level of activity observed [35]–[37]. In tobacco, expression in pollen from the CaMV 35S promoter has been reported, albeit at a lower level than other promoters, e.g., from the *nos* or *lat52* genes [36], [38]. The higher level of DHAR activity in pollen of D_Ta_ and D_Nt_ plants indicates that the CaMV 35S promoter used is active during pollen development. To follow the fate of expression from the CaMV 35S promoter in pollen during its subsequent growth through the style and in ovules following fertilization, luciferase expression from tobacco plants containing a 35S::*Luc* transgene was used as a proxy for DHAR expression. Significant luciferase expression was detected in mature pollen of D_Ta_ plants whereas wild-type pollen, style, or ovules had no detectable luciferase expression (Table 1). Two days following pollination, luciferase expression was also detected in the style (excluding the stigma where the pollen grains were present) and in ovules as well (Table 1). Similar results were obtained when pollen from 35S::*Luc* tobacco was used in crosses with D_Ta_ or D_Nt_ plants (Table 1). These results indicate that the CaMV 35S promoter used is active during pollen development in tobacco. These results also indicate that, like luciferase, DHAR accompanies growth of the pollen tube through the style and is likely delivered to the egg following fertilization. Any pollen-mediated increase in Asc levels in the fertilized egg would be expected to be greatest at the earliest stage of embryogenesis. As discussed below, increasing Asc in the ovules was effective in inducing twinning only during early embryo development.

**Table 1:** Transmission of pollen cytoplasm to fertilized eggs.

Female	Male	Organ assayed	Days after pollination (DAP)	Luciferase activity (Light units/mg protein/min)
Wild-type		pollen	0	0
		style	0	0
		ovules	0	0
	35S::*Luc*	pollen	0	1.15×10^9^±1.21×10^7^
Wild-type	35S::*Luc*	style	2	9.53×10^8^±6.44×10^7^
	35S::*Luc*	ovules	2	2.21×10^7^±3.96×10^6^
D_Ta_	35S::*Luc*	style	2	3.60×10^8^±2.10×10^6^
	35S::*Luc*	ovules	2	3.30×10^6^±1.92×10^6^
D_Nt_	35S::*Luc*	style	2	4.82×10^8^±1.85×10^7^
	35S::*Luc*	ovules	2	2.35×10^7^±3.66×10^6^

### Polyembryony induced by DHAR results from monozygotic twinning

**Figure 4:**
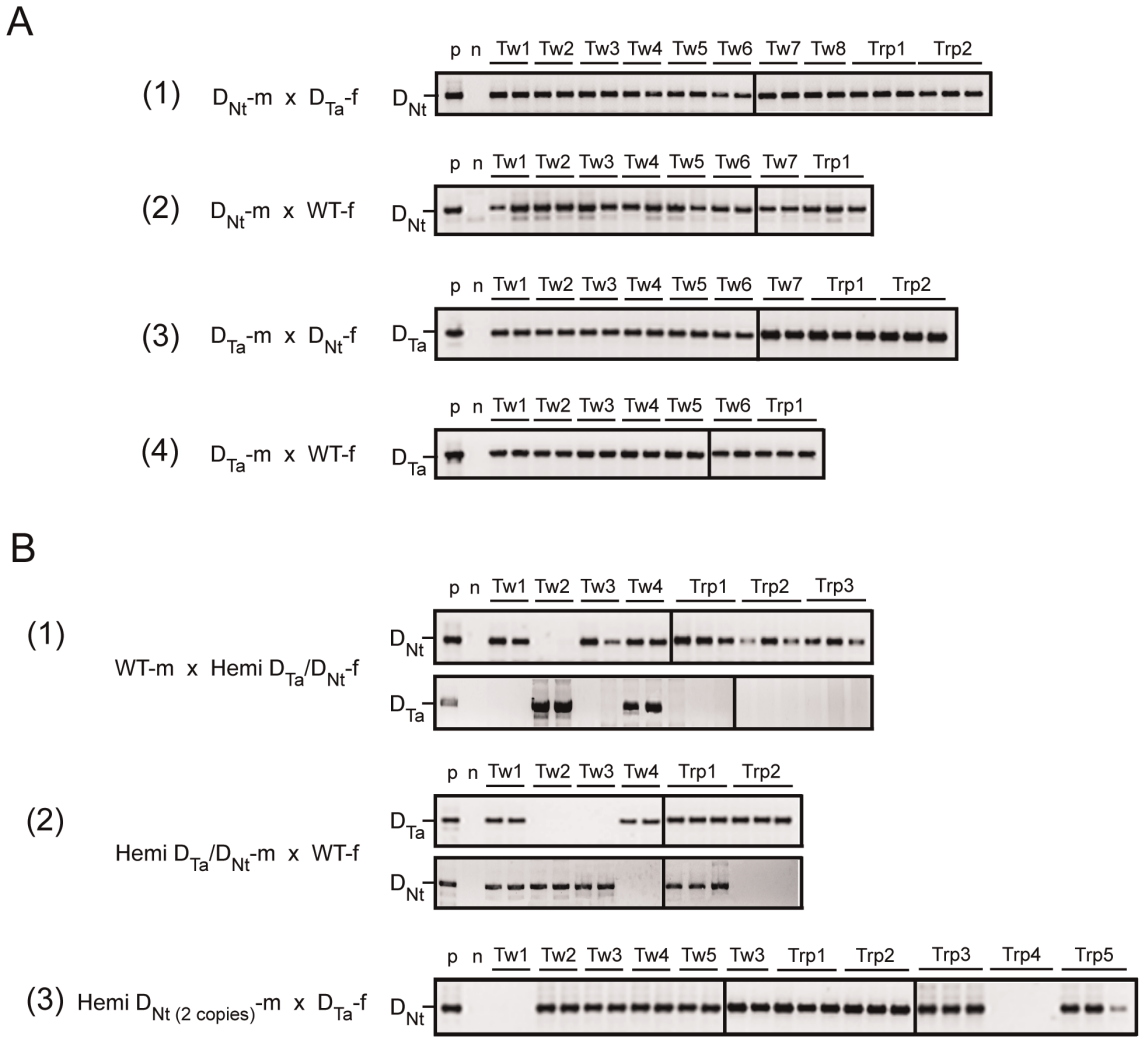
Analysis of the genetic relationship among twin and triplet progeny. (A) Inheritance of the D_Ta_ or D_Nt_ transgenes when transmitted through pollen (m) or egg (f) homozygous for the transgene. Inheritance of the D_Nt_ transgene in twin (Tw) and triplet (Trp) progeny from D_Nt_ pollen (D_Nt_-m) when used to fertilize (1) D_Ta_ or (2) WT flowers (WT-f). Inheritance of the D_Ta_ transgene in twin and triplet progeny from D_Ta_ pollen (D_Ta_-m) when used to fertilize (3) D_Nt_ or (4) WT flowers. (B) Inheritance of the D_Ta_ or D_Nt_ transgenes when transmitted through pollen (m) or egg (f) hemizygous for each transgene. (1) Inheritance of the D_Nt_ and D_Ta_ transgenes in twin and triplet progeny from eggs hemizygous for the D_Ta_ and D_Nt_ transgenes (D_Ta_/D_Nt_-f) when fertilized by WT pollen (WT-m). (2) Inheritance of the D_Nt_ and D_Ta_ transgenes in twin and triplet progeny from pollen hemizygous for the D_Ta_ and D_Nt_ transgenes (D_Ta_/D_Nt_-m) when used to fertilize WT flowers. (3) Inheritance of the D_Nt_ transgene in twin and triplet progeny from pollen hemizygous for D_Nt_ transgene present in 2 copies in the genome (D_Nt(2 copies)_-m) when used to fertilize D_Ta_ flowers (D_Ta_-f). p: PCR of tobacco containing the appropriate DHAR transgene to serve as a positive control. n: PCR of wild type tobacco to serve as a negative control.

Polyembryony can arise through apomixis, i.e., the generation of embryos from maternal cells alone (e.g., parthenogenesis, apogamy, or apospory) or through twinning, i.e., the generation of multiple embryos from the fertilized egg or embryonic cells. Paternal inheritance is absent in apomitic embryos but present in true twins. To determine whether the observed polyembryony was a consequence of apomixis or represented twinning, genetic analysis of the progeny from polyembryonic seeds was performed to determine paternal inheritance. All progeny of polyembryonic seeds resulting from a D_Nt_-m×D_Ta_-f cross or a D_Nt_-m×WT-f cross inherited the pollen-transmitted D_Nt_ transgene (Fig. 4A). These results exclude the possibility of apomixis and suggest that the observed polyembryony results from twinning. These results are also consistent with the observation that the polyembryony from a D_Nt_-m×WT-f cross or D_Ta_-m×WT-f cross occurred following fertilization since WT ovules would not be expected to produce twins until after fertilization by D_Nt_ pollen. In some cases, fertilization is required to induce apomixis. To rule out that apomixis was induced when the D_Ta_ transgene was pollen-transmitted, the presence of the D_Ta_ transgene in polyembryonic progeny resulting from a D_Ta_-m×D_Nt_-f cross or a D_Ta_-m×WT-f cross was determined. All polyembryonic progeny inherited the D_Ta_ transgene as transmitted through the male gametes (Fig. 4A), ruling out the generation of adventitious, non-zygotic embryos that might have been induced following fertilization by D_Ta_-pollen.

It was possible that the DHAR-induced polyembryonic seed resulted from fusion of independently fertilized ovules as observed in maize [39] or dizygotic twinning, i.e., the independent fertilization of multiple eggs within a single ovule by multiple male gametes [40], [41]. To examine this possibility, the transgene inheritance in polyembryonic progeny from a cross between a WT-m and a hemizygous D_Ta_/D_Nt_-f or between a hemizygous D_Ta_/D_Nt_-m and a WT-f was determined. The uniform inheritance of either the D_Nt_ transgene or the D_Ta_ transgene from a hemizygous D_Ta_/D_Nt_-f parent in the polyembryonic progeny of a seed would rule out fusion of independently fertilized ovules. Similarly, the uniform inheritance of either the D_Nt_ transgene or the D_Ta_ transgene from a hemizygous D_Ta_/D_Nt_-m parent in the polyembryonic progeny of a seed would rule out dizygotic twinning. Progeny from a cross between a WT-m and a hemizygous D_Ta_/D_Nt_-f were identical in their transgene inheritance as were progeny from a cross between a hemizygous D_Ta_/D_Nt_-m and a WT-f (Fig. 4B), i.e., the inheritance or lack of inheritance of either the D_Ta_ or D_Nt_ transgene was uniform for all progeny within a given polyembryonic seed. Progeny were also identical in their inheritance of the D_Nt_ transgene from male gametes hemizygous for this transgene in a cross with a D_Ta_ female (Fig. 4B). These results ruled out the possibility of fusion of independently fertilized ovules or dizygotic twinning, supporting the notion that the progeny arose from a single zygote.

**Figure 5:**
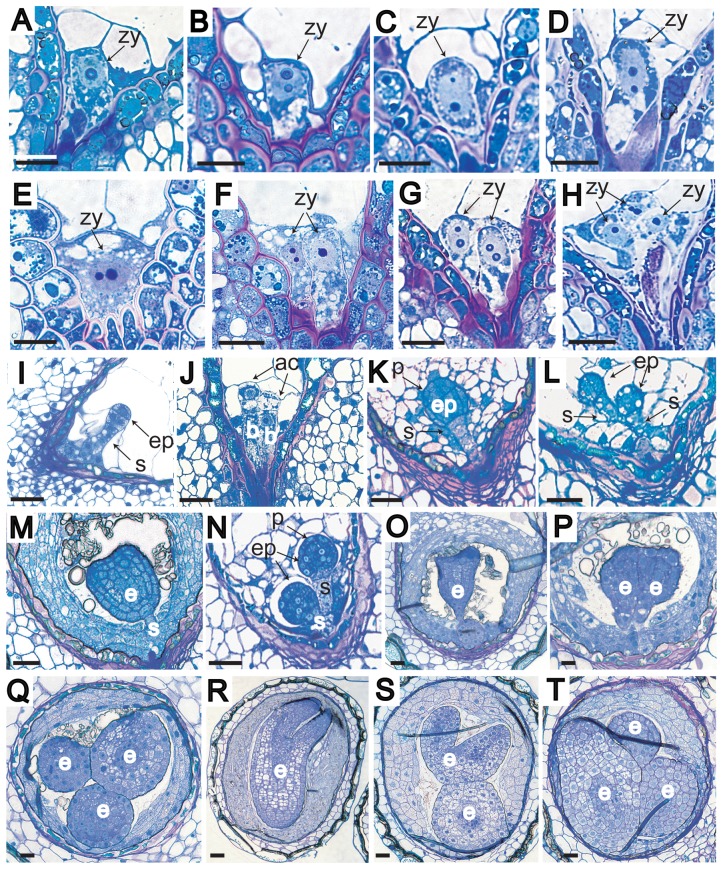
Development of twin and triplet embryos in polyembryonic seed. **A–D**, Developing WT zygotes showing growth prior to the first zygotic division. **E–H**, Developing zygotes in D_Ta_ ovules showing (**E**) horizontal position of nucleoli prior to the first zygotic division, (**F**) presence of twin zygotes in adjacent proximity with vertical position of nucleoli in one of the zygotes, (**G**) presence of twin zygotes in adjacent proximity with vertical position of nucleoli in both zygotes, (**H**) presence of triplet zygotes. **I–J**, Developing embryos at 5 DAP from (**I**) WT and (**J**) D_Ta_ plants showing twin proembryos at the same developmental stage. **K–L**, Developing embryos at 6 DAP from (**K**) WT and (**L**) D_Ta_ plants. Cells of the protoderm are evident in the embryo proper of the WT embryo not present in the twin D_Ta_ embryos as the latter are delayed in their development. **M–N**, Developing embryos at 8 DAP from (**M**) WT and (**N**) D_Ta_ plants. **O–Q**, 10 DAP developing embryos from (**O**) WT and (**P**) D_Ta_-double and (**Q**) D_Ta_-triple embryo seed. **R–T**, 12 DAP developing embryos from (**R**) WT and (**S**) D_Ta_-double and (**T**) D_Ta_-triple embryo seed. ac, apical cell; b, basal cell; e, embryo; ep, embryo proper; p, protoderm; s, suspensor; zy, zygote. Scale bars represent 50 µm.

Monozygotic twinning, i.e., twinning that occurs during the first zygotic division, delays embryo development by the time required for the first zygotic division. However, as the twin embryos would be delayed equally, they would exhibit a similar rate of development. To examine whether this may account for the DHAR-induced polyembryony, the development of twin embryos was compared to that of WT embryos. Two or more zygotes were observed in developing seed from DHAR-overexpressing plants (Fig. 5F–H) compared to only one zygote in WT seed (Fig. 5A–D). At 5 days after pollination (DAP), two proembryos were observed in DHAR-overexpressing seed (Fig. 5J). Each proembryo was composed of an apical to two-celled proembryo and a basal cell, indicating that twinning occurred prior to the transverse division of the zygote. Both proembryos were at a similar developmental stage, arguing against the possibility that the twin arose from the embryo-proper or suspensor of the primary embryo. Both embryos were developmentally delayed relative to WT embryos which were at quadrant to octant (Fig. 5I). Similarly, when WT embryos were at mid-globular (Fig. 5K) or transition (Fig. 5M) stages, twin DHAR-overexpressing embryos, each with a distinct suspensor, were at early-globular (Fig. 5L) or mid-globular (Fig. 5N) stages, respectively, supporting the conclusion that embryo development was delayed as a consequence of the twinning event during the first zygotic division. Analysis at subsequent stages revealed the continued development of double and triple progeny within a seed (Fig. 5P–Q, S–T) relative to a single embryo in WT seed (Fig. 5R). Seedlings from polyembryonic seeds were of similar size (Fig. 1A–H), supporting the notion that their development had initiated simultaneously.

Determination of the embryo cell number (estimated from the number of cells in median, longitudinal sections) demonstrated that WT embryos contained an average of 75 cells (representing 6.2 cell divisions) at 6.5 DAP (Table 2) whereas twin DHAR-overexpressing embryos contained an average of 26.5 cells (4.7 cell divisions), a delay of 1.5 cell divisions. At 8 DAP, WT embryos contained 212 cells (7.7 cell divisions) whereas twin embryos contained 73 cells (6.2 cell divisions). These data suggest that twin embryos had undergone 1.5 fewer cell divisions than WT embryos, consistent with the delayed development associated with monozygotic twinning.

**Table 2:** Cell number of twinned embryos relative to single embryos.

Embryo type	Days after pollination	Embryo diameter (cell number)	Embryo size (total cell number)	Number of cell divisions
Monoembryonic	6.5	5.3±0.7	75	6.2
Polyembryonic	6.5	3.7±0.8	26.5	4.7
Monoembryonic	8	7.4±0.7	212	7.7
Polyembryonic	8	5.2±0.9	73	6.2

### Ascorbic acid phenocopies DHAR-induced twinning

**Figure 6:**
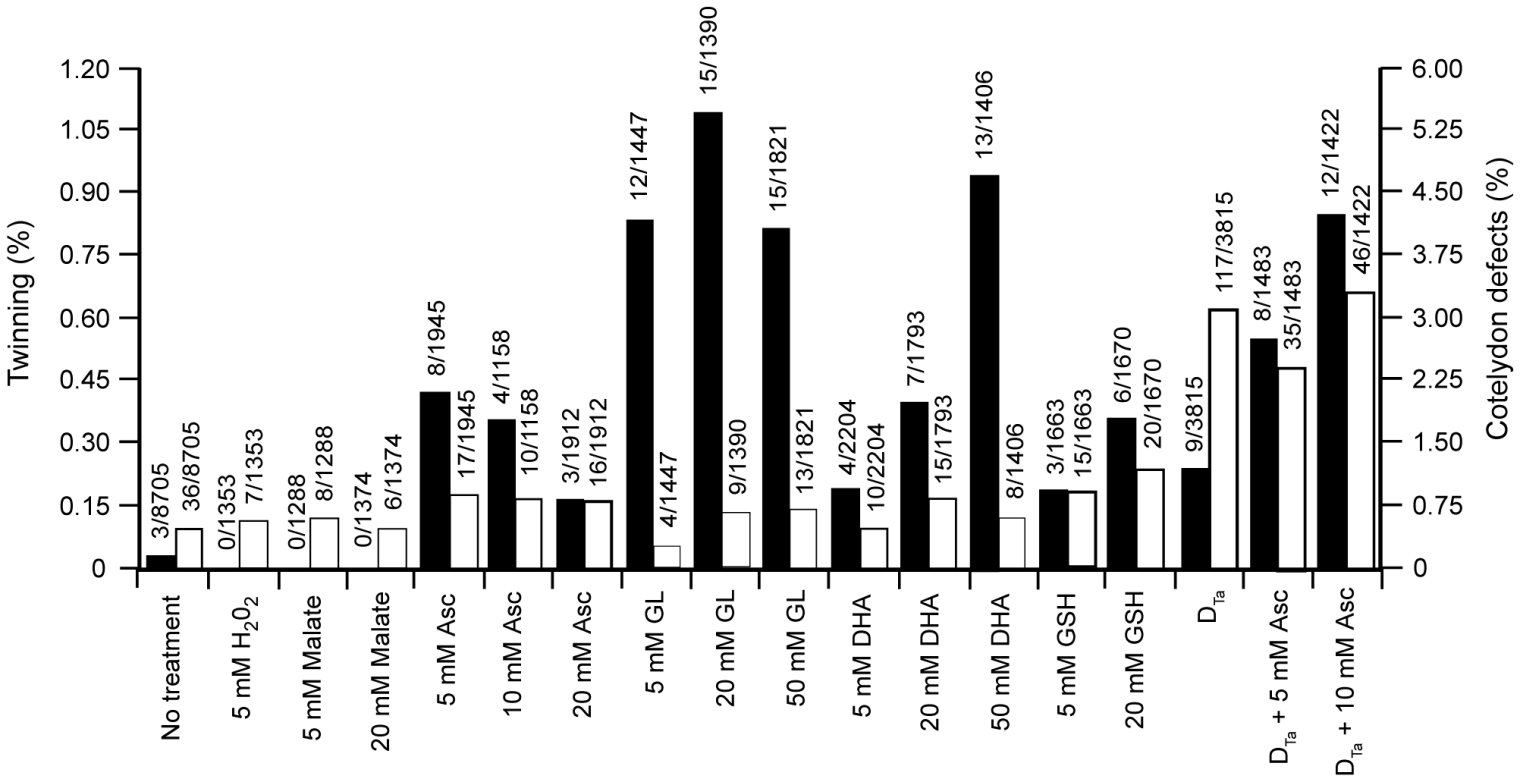
Twinning is induced by Asc or compounds involved in Asc biosynthesis or recycling. Frequency of twinning and polycotyly in progeny of WT ovaries injected with (**A**) Asc, GL, dehydroascorbate (DHA), glutathione (GSH), H_2_O_2_, or malate at the concentrations indicated on the day of pollination. The histograms represent the frequency of twinning (black bars, left scale) and cotyledon abnormalities (i.e., polycotyly or single fused cotyledon)(white bars, right scale) observed following germination.

Overexpression of DHAR results in an increase in the Asc pool size [28]. To test directly whether an increase in Asc was responsible for the DHAR-induced twinning, Asc, at concentrations similar to those present in plants [42], was injected into WT ovaries on the day of pollination. The frequency of twinning in progeny of WT plants increased substantially following the injection of Asc (Figs. 1I–J and 6) or its immediate precursor, L-galactono-1,4-lactone (GL) (Fig. 6) which is known to increase the Asc pool size [43], [44]. Injection of Asc or GL also further increased the frequency of twinning following injection into ovaries of D_Ta_ plants (Fig. 6). Injection of dehydroascorbate (DHA), which increases the Asc pool size [44] following its reduction to Asc by DHAR in a glutathione-requiring reaction, induced twinning as did injection with glutathione (Fig. 6). Injection of 5 mM Asc resulted in more polyembryony than did higher levels of Asc, perhaps due to a threshold effect beyond which further increases in Asc have no additional effect. It is also possible that injecting high concentrations of Asc limits its ability to promote polyembryony through its effect on the Asc redox state of the apoplast. Asc is normally present in the apoplast at only very low concentrations as DHA is the dominant form [27]. Injection of Asc would transiently increase the Asc redox state of the apoplast in addition to increasing the intracellular Asc concentration. Injection of Asc at higher concentrations than 50 mM caused death of the ovaries indicating that it has toxic effects not seen with GL or DHA at similar concentrations. Injection of Asc on the day of pollination induced only a slight increase in cotyledon defects (Fig. 6), demonstrating that the effect of Asc on twinning can be temporally separated from its effect on polycotyly. Injection of Asc later in embryo development, however, did increase polycotyly substantially (see below). No increase in the frequency of twinning was observed in ovaries injected with water, H_2_O_2_, organic acids such as malate (Fig. 6) or citric acid, or α-tocopherol (data not shown). These data support the conclusion that twinning is induced by Asc or compounds known to increase intracellular Asc levels [44]–[49].

**Figure 7:**
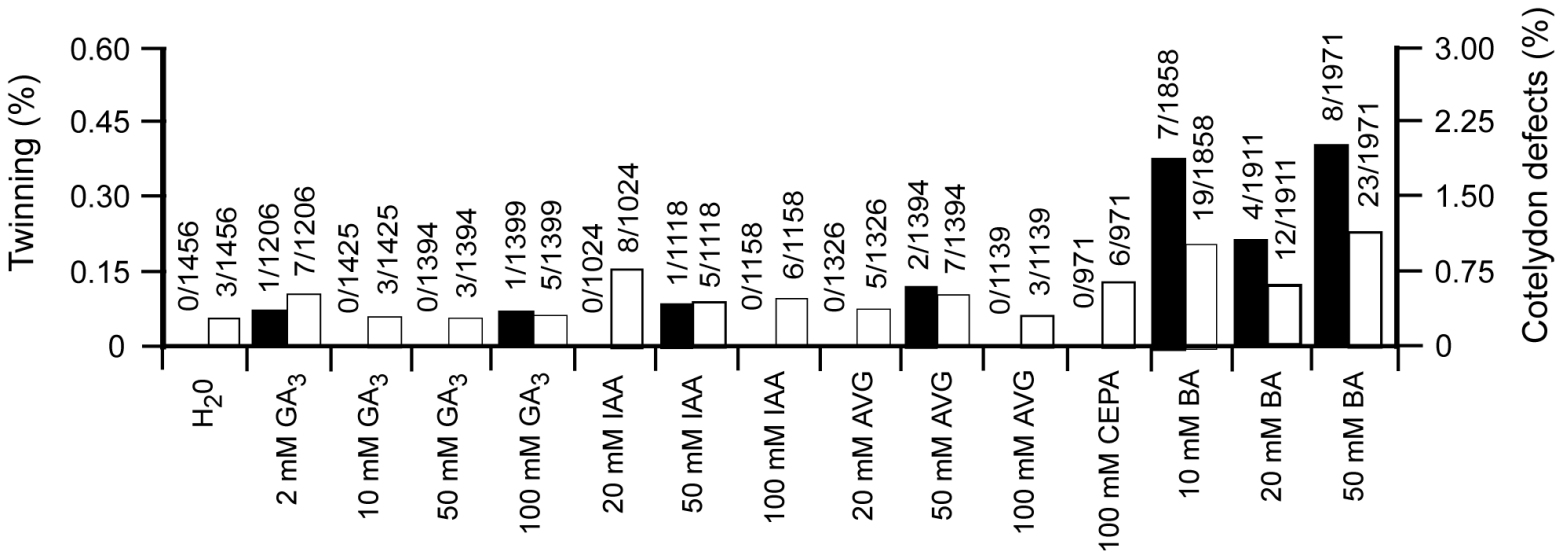
Hormonal control of twinning. Frequency of twinning and polycotyly in progeny of WT ovaries injected with gibberellic acid (GA_3_), indole acetic acid (IAA), aminoethoxyvinylglycine (AVG), 2-chloroethylphosphonic acid (CEPA) that produces ethylene, or the cytokinin, benzyladenine (BA) at the concentrations indicated on the day of pollination. The histograms represent the frequency of twinning (black bars, left scale) and cotyledon abnormalities (i.e., polycotyly or single fused cotyledon)(white bars, right scale) observed following germination.

Auxin and cytokinin play important roles in regulating cell division and changes to the auxin gradient in the female gametophyte can alter the number of egg cells per embryo sac [41]. The involvement of Asc in the synthesis of some hormones raised the possibility that perturbation in hormone levels in the zygote may be involved in the Asc-induced twinning. To examine whether altered levels of hormones might influence twinning, gibberellic acid (GA_3_), auxin (IAA), cytokinin (BA), the ethylene-releasing compound 2-chloroethylphosphonic acid (2-CEPA), or aminoethoxyvinylglycine (AVG), an inhibitor of ethylene biosynthesis was injected into WT ovaries. Only BA resulted in a consistent increase in the frequency of polyembryony and polycotyly (Fig. 7). Injection of a combination of BA and Asc did not increase polyembryony above that observed for BA (data not shown), suggesting that Asc and cytokinin may affect the same developmental step leading to twinning.

### Ascorbic acid-induced twinning is developmentally limited to the first two days after pollination

**Figure 8:**
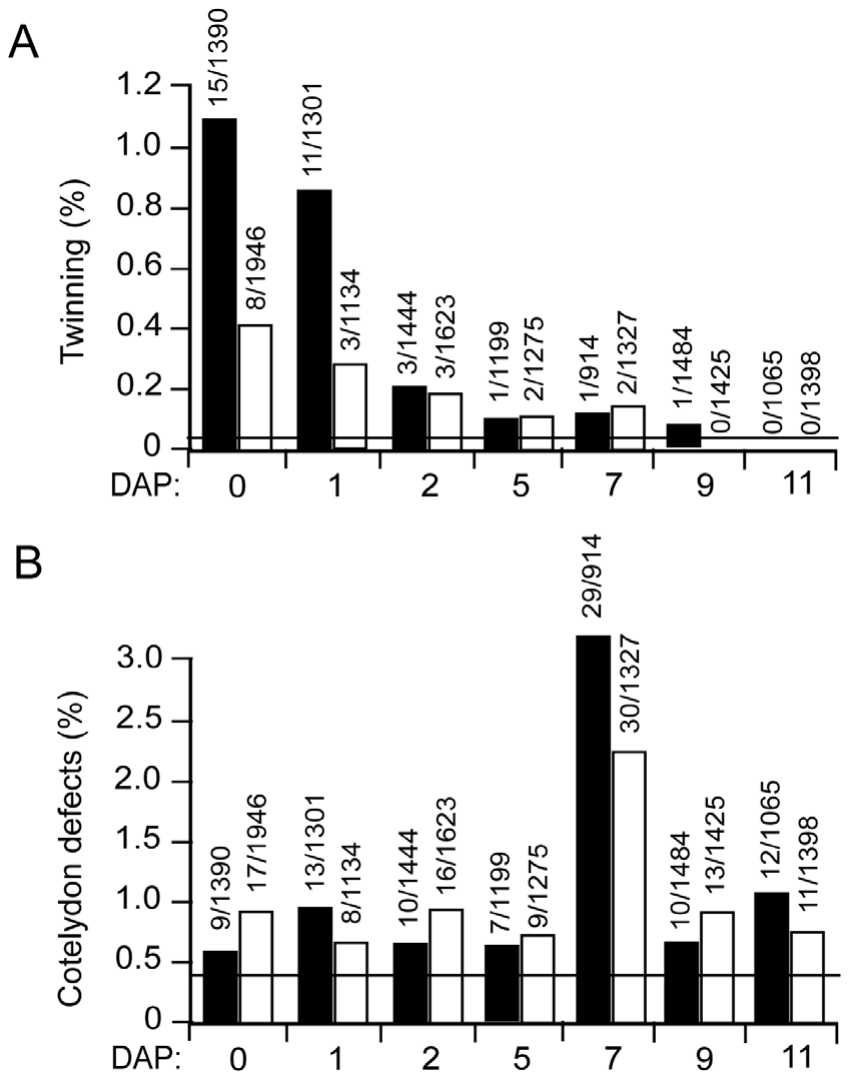
Determination of the developmental stage receptive to Asc-induced twinning and polycotyly. 5 mM Asc (white bars) or GL (black bars) was injected into WT ovaries the day of pollination or on days after pollination as indicated. **A**, The frequency of twinning and (**B**) polycotyly observed in the resulting progeny. The horizontal line represents the spontaneous frequency of polyembryony or cotyledon abnormalities.

A study of the timing of fertilization in tobacco reported no evidence of fertilization by 48 hours after pollination (HAP) and only 50% egg cells showing various stages of fusion of the egg and sperm nuclei at 54 HAP [50]. Zygotic division did not occur prior to 84 HAP [50]. If Asc induces twinning, its effect should be limited to the period prior to the first zygotic division following fertilization. Twinning was induced substantially when GL or Asc was injected within 0–2 DAP but lower rates were observed when either was injected after 2 DAP (Fig. 8A), consistent with its role in regulating the first zygotic division but not subsequent cell divisions until cotyledon development. GL or Asc injected on the day of pollination did lead to a small increase in cotyledon defects (Fig. 8B) as was also observed in Fig. 6 but the highest frequency of polycotyly was observed when GL or Asc was injected at 7 DAP (Fig. 8B), the developmental stage representing the transition from globular to heart stage during which cotyledon formation initiates (Fig. 5K and M, respectively). These data demonstrate that early embryo development is sensitive to Asc-induced twinning and polycotyly, consistent with the notion that Asc alters zygotic division and apical patterning during cotyledon initiation, respectively. In contrast to the *sus* or *rsp* mutants of Arabidopsis in which the suspensor undergoes inappropriate cell divisions and takes on some characteristics of embryo-proper cells [51], [52], suspensor development was unaffected by Asc (Fig. 5J, L, N), indicating that the signaling between the embryo-proper and suspensor cells was maintained.

### Cytoskeleton altering agents phenocopy ascorbic acid-induced polyembryony

The changes in cell polarity, and thus cell division, caused by Asc during embryo development suggested possible alterations to the elements of the cytoskeleton, i.e., microtubules or microfilaments, which determine the orientation or perhaps rate of cell division. Pharmacologically perturbing the cytoskeleton during zygotic cell division might also be expected result in polyembryony. To investigate this, WT ovaries were injected at 48 hours after pollination (HAP) with taxol, which stabilizes microtubules and thus perturbs the bundling of microtubules involved in formation of the spindle poles and the preprophase band (PPB) that marks the division site [53] Progeny from taxol-treated ovaries exhibited a 15-fold increase in polyembryony (0.74%; 13/1747) relative to that observed in untreated ovaries (0.05%; 1/1846). Taxol injected at 54 HAP, i.e., following fertilization but prior to the first zygotic division [50], resulted in an 11-fold increase in polyembryony (0.56%; 7/1255). Cytochalasin B, which inhibits actin polymerization resulting in misorientation of new cell walls [54], increased polyembryony nearly 5-fold over the basal frequency (0.24%; 3/1248) when injected at 48 HAP or 3-fold (0.14%; 2/1461) when injected at 54 HAP.

Consistent with notion that Asc may affect aspects of cytokinesis was the central position of the nucleus (with transverse nucleoli) surrounded by vacuoles in a non-elongated, DHAR-overexpressing zygote (Fig. 5E). An increase from one nucleolus in the egg cell to two vertically positioned nucleoli in the fertilized zygote has been described and has been suggested to represent karyogamy [55]. Such a position of the nucleus is in contrast to WT zygotes in which the nucleus (with vertically positioned nucleoli) is typically present in the apical region of an elongated cell above a vacuolated basal region (Fig. 5A–D). Subsequent divisions of horizontally-positioned, twin zygotes show a wild-type asymmetric position of the nucleus (with vertically positioned nucleoli) above a vacuolated basal region in elongated cells (Fig. 5F–G).

## Discussion

We have shown that ascorbic acid promotes polyembryony and polycotyly when its level is elevated either by the activity of DHAR, which increases the Asc pool size as a consequence of its recycling function [25]–[28], or through direct application to ovules. Apomixis was ruled out as the basis for the polyembryony because progeny of polyembryonic seed inherited pollen-transmitted genes which does not occur in apomictic progeny. We conclude that Asc-induced polyembryony is a result of monozygotic twinning because 1) polyembryony was induced only when Asc was injected prior to the first zygotic division; 2) two or more zygotes were observed in polyembryonic seed; 3) twin embryos with normal suspensors developed at the same rate and were developmentally delayed with respect to WT embryos; and 4) twin progeny in DHAR-induced polyembryonic seed were genetically identical. These observations suggest that Asc affects the first zygotic division and, in the case of triplet progeny, the subsequent round of division as well.

In addition to DHAR or ascorbic acid, itself, compounds that affect the pool size of ascorbic acid also induced polyembryony. For example, feeding experiments with the immediate precursor of ascorbic acid, L-galactono-1,4-lactone (GL), increased the Asc pool size [43], [44] and induced polyembryony to a high level. Similar observations were made with dehydroascorbate (DHA), the substrate for DHAR, which also increases the Asc pool size [44] and to lesser extent with glutathione which is used by DHAR to reduce DHA to Asc. Organic acids unrelated to ascorbic acid or its biosynthesis had no effect on polyembryony. The specificity of DHAR and the effect that ascorbic acid or compounds that affect its pool size had on polyembryony strongly implicate ascorbic acid as responsible for the twinning.

Changes to the auxin gradient within the female gametophyte can alter the number of egg cells per embryo sac and consequently, the number of fertilization events by independent pollen tubes entering the ovule [41]. However, as the auxin gradient functions by regulating cell fate specification within the embryo sac at cellularization [41], the polyembryony resulting from transmission of a DHAR transgene by male gametes or following delivery of ascorbic acid to an ovary the same day as fertilization is inconsistent with an ascorbic acid-mediated change in the auxin gradient generating multiple egg cells as is the observation that twin progeny in DHAR-induced polyembryonic seed were genetically identical.

Although increasing the expression of DHAR in the male or female parent revealed that polyembryony was induced by paternal or maternal inheritance of a DHAR transgene, increasing DHAR expression throughout the female parent resulted in a greater induction of polyembryony than did inheritance of the same transgene through male gametes. Suppression of DHAR expression in tissues other than the style and ovules also resulted in a substantially lower level of polyembryony than when its expression was elevated throughout the plant. These observations suggest that the elevated expression of DHAR in maternal tissues likely contributes to the increase in the Asc pool size in the ovule. Nevertheless, the observation that inheritance of a DHAR transgene through male gametes can increase polyembryony demonstrates that confining expression of the transgene to embryonic tissues is sufficient to influence the rate of twinning.

Embryogenesis begins with an asymmetric transverse division of the zygote, resulting in an elongated basal cell that initiates acquisition of suspensor identity and a smaller nonvacuolated apical cell that specifies into the embryo proper. The asymmetric division resulting in daughter cells of different sizes or cytoplasmic determinants may be required for the subsequent specification of the apical and basal cells [56], [57]. Cell specification may also require positional information, e.g., contact of the basal cell with the maternal tissue [7], [58], [59]. Asc-induced twinning suggests that the plane of cytokinesis during early embryo development is altered resulting in symmetric division, a loss of positional information following the first zygotic division, and a lack of cell specification of the daughter cells that then develop into genetically identical twins. This possibility is supported by the ability of cytoskeleton-perturbing agents to phenocopy Asc-induced polyembryony. Treatment with taxol is known to perturb formation of the preprophase band that marks the division site and treatment with cytochalasin B results in misorientation of new cell walls [54], consistent with the notion that the control of the plane or rate of cell division during the first zygotic division may be important to avoid twinning. Of the phytohormones tested, only cytokinin was able to induce polyembryony, suggesting that this hormone may influence the positioning of the plane or rate of cell division during the first zygotic division, consistent with its role in regulating cell division [60], [61]. Kinetin has been shown to cause the longitudinal orientation of cortical microtubules resulting in the deposition of cellulose microfibrils in the same orientation [62], [63] and demonstrating a potential role for this hormone in the orientation of cell division during embryogenesis.

Asc has been implicated in promoting G1 to S phase progression as was shown in cells within the QC of roots where cells remain in G1 longer than cells surrounding the QC [16]–[20], [64], [65]. The observation that the development of twin embryos is temporally delayed relative to WT embryos suggests that Asc does not induce a premature initiation of the first zygotic division prior to the correct positioning of the plane of cell division. Our observations do suggest, however, that one or more steps involved in cytokinesis or the rate of cell division are affected by Asc which are revealed during critical developmental stages including the first zygotic division and specification of cotyledon-forming fields. Although these two stages during embryo development may be particularly sensitive to Asc, it is equally plausible that they simply represent developmental stages in which perturbations in cell division are most easily observed. The maximum rate of twinning resulting from increased DHAR expression was just over 3% and most stages of embryo development may be able to tolerate this level of alterations to cell division without resulting in a readily apparent phenotype. The identification of the step(s) during cell division affected by Asc will be necessary in future work in order to understand fully the role that Asc plays in cell division.

## Materials and Methods

### Plant growth conditions

Full-length wheat DHAR (D_Ta_) cDNA (Accession number: AY074784) and tobacco DHAR (D_Nt_) cDNA (Accession number: AY074787) under the control of the CaMV 35S promoter in the binary vector, pBI101, were used to generate D_Ta_-overexpressing and D_Nt_-silenced tobacco (*N. tabacum*, cv. Xanthi), respectively, using *Agrobacterium tumefaciens* as described previously [25], [26]. pBI101 containing the firefly luciferase (*Luc*) was also used to generate tobacco expressing luciferase. Transgenic tobacco plants were grown in a greenhouse supplied with charcoal-filtered air. Compounds were injected into ovaries in a 50 µl volume on the day of pollination or on subsequent days as indicated. Taxol and cytochalasin B were used at a concentration of 25 µM. Clearing intact cotyledons of chlorophyll was performed using 95% ethanol for 2 hr with one change of solution.

### Microscopy

Seeds were germinated on 0.8% agar containing one-half strength MS salts containing 1% sucrose. Images were collected using a dissecting microscope. Ovaries containing developing seeds were fixed in FAA (50% ethanol, 5% acetic acid, 3.7% formaldehyde) at 4°C and dehydrated through a graded ethanol series to 100%. The samples were embedded in Paraplast or in resin. Sections were stained with Toluidine Blue O and images were collected using a compound microscope. Images of ovules were collected using a dissecting microscope.

### DHAR enzyme assay

DHAR activity was assayed essentially as described [66]. Soluble protein was extracted from tobacco ground in liquid nitrogen before grinding in extraction buffer (50 mM Tris-HCl pH 7.4, 100 mM NaCl, 2 mM EDTA, 1 mM MgCl_2_) and centrifuging twice at 13,000 rpm for 5 min to remove cell debris. Protein concentration was determined as described [67]. DHAR activity was assayed from an equal amount of protein in 50 mM K_2_HPO_4_/KH_2_PO_4_ pH 6.5, 0.5 mM DHA, and 1 mM GSH and activity was followed by an increase in absorbance at 265 nm.

### DNA isolation

Leaf tissue was ground in liquid nitrogen to fine powder, then, mixed with extraction buffer (containing 100 mM Tris-HCl pH 9.0, 200 mM NaCl, 1% sarcosyl, 20 mM EDTA, and 10 µl/ml β–mercaptoethanol). After extracted with equal volume of phenol/chloroform, RNA was removed from the supernatant by precipitating with 2 M LiCl. Equal volume of isopropanol was added to the RNA-depleted supernatant to pellet the DNA.

### Polymerase chain reaction

Amplification of transgenes was performed in 25 µl reactions containing 1× PCR buffer, 1 u HotStarTaq DNA polymerase (Qiagen Inc, Valencia CA, U.S.A.), 250 µM dNTP, 10 µM forward and reverse primers, and 50 ng genomic DNA. Reactions were carried out using the following conditions: 95°C/15 min (1 cycle); 95°C/30 sec, 56°C/30 sec for the tobacco DHAR gene (62°C/30 sec for the wheat DHAR gene), 72°C/1.5 min (35 cycles); and extension at 72°C for 1.5 min; and a final extension at 72°C/5 min (1 cycle). The upstream primer is 5′-ACTGACGTAAGGGATGACGCA-3′, which anneals within the CaMV 35S promoter region. The downstream primers are 5′-GCGAAACAACGGGATTATAATTATG-3′ (tobacco DHAR gene) and 5′GGATCCAGGGGCTTACGGGTTCACTTTC-3′ (wheat DHAR gene), respectively.

### Statistical analysis of PCR results

Paternal or maternal inheritance using PCR was performed on multiple twin/triplet embryos. All progeny within a seed exhibited similar paternal inheritance, ruling out apomixis. The possibility of multiple fertilization events of a polyembyro-containing ovule or the post-fertilization fusion of ovules was considered highly unlikely as Mendelian inheritance of paternal genes would be expected. The probability that twin progeny resulting from independent fertilization events exhibit similar paternal inheritance (i.e., either both inherit or both fail to inherit) of a pollen-transmitted transgene from a hemizygous parent is one out of two, i.e., a 50% chance that the twin seedlings are genetically identical. For triplet embryos, there is a 25% chance that they are genetically identical if from independent fertilization events. The probability that independent fertilization was responsible for twin embryos in 4 seed and triplet embryos in 3 seed and resulted in uniform inheritance of a pollen-transmitted transgene from a hemizygous parent is (1/2)^4^×(1/4)^3^ = 1/1024. Therefore, there is a 99.9% probability that the uniform inheritance resulted from twinning from a single zygote and not from multiple fertilization event.

### Luciferase assay

Tobacco tissue was frozen in liquid nitrogen and ground in luciferase assay buffer (25 mM Tricine pH 8, 5 mM MgCl_2_, 0.1 mM EDTA supplemented with 33.3 mM DTT, 270 µM coenzyme A, 500 µM ATP). Following centrifugation, the extract was assayed for luciferase activity following injection of 0.5 mM luciferin using a Monolight 2010 Luminometer (Analytical Luminescence Laboratory). Triplicate samples were assayed and the average value is reported.
